# Clinicians’ and Patients’ Perspectives on Hypertension Care in a Racially and Ethnically Diverse Population in Primary Care

**DOI:** 10.1001/jamanetworkopen.2023.0977

**Published:** 2023-02-28

**Authors:** Julie C. Lauffenburger, Renee A. Barlev, Rasha Khatib, Nicole Glowacki, Alvia Siddiqi, Marlon E. Everett, Michelle A. Albert, Punam A. Keller, Lipika Samal, Kaitlin Hanken, Ellen S. Sears, Nancy Haff, Niteesh K. Choudhry

**Affiliations:** 1 Division of Pharmacoepidemiology and Pharmacoeconomics, Department of Medicine, Brigham and Women’s Hospital, Harvard Medical School, Boston, Massachusetts; 2 Center for Healthcare Delivery Sciences (C4HDS), Department of Medicine, Brigham and Women’s Hospital, Harvard Medical School, Boston, Massachusetts; 3 now at Vytalize Health, Hoboken, New Jersey; 4 Advocate Aurora Research Institute, Advocate Aurora Health, Downers Grove, Illinois; 5 Enterprise Population Health, Advocate Aurora Health, Downers Grove, Illinois; 6 Advocate Heart Institute, Advocate Aurora Health, Chicago, Illinois; 7 Center for the Study of Adversity and Cardiovascular Disease (NURTURE Center), Division of Cardiology of Medicine (Cardiology), University of California, San Francisco, San Francisco; 8 Tuck School of Business, Dartmouth College, Hanover, New Hampshire; 9 Division of General Internal Medicine, Department of Medicine, Brigham and Women’s Hospital, Harvard Medical School, Boston, Massachusetts

## Abstract

**Importance:**

Hypertension control remains suboptimal, particularly for Black and Hispanic or Latino patients. A need exists to improve hypertension management and design effective strategies to efficiently improve the quality of care in primary care, especially for these at-risk populations. Few studies have specifically explored perspectives on blood pressure management by primary care providers (PCPs) and patients.

**Objective:**

To examine clinician and patient perspectives on barriers and facilitators to hypertension control within a racially and ethnically diverse health care system.

**Design, Setting, and Participants:**

This qualitative study was conducted in a large urban US health care system from October 1, 2020, to March 31, 2021, among patients with a diagnosis of hypertension from a racially and ethnically diverse population, for a range of hypertension medication use hypertension control, as well as practicing PCPs. Analysis was conducted between June 2021 and February 2022 using immersion-crystallization methods.

**Main Outcomes and Measures:**

Perspectives on managing blood pressure, including medication adherence and lifestyle, considerations for intensification, and experiences and gaps in using health information technology tools for hypertension, were explored using semistructured qualitative interviews. These cycles of review were continued until all data were examined and meaningful patterns were identified.

**Results:**

Interviews were conducted with 30 participants: 15 patients (mean [SD] age, 58.6 [16.2] years; 10 women [67%] and 9 Black patients [60%]) and 15 clinicians (14 PCPs and 1 medical assistant; 8 women [53%]). Eleven patients (73%) had suboptimally controlled blood pressure. Participants reported a wide range of experiences with hypertension care, even within the same clinics and health care system. Five themes relevant to managing hypertension for racially and ethnically diverse patient populations in primary care were identified: (1) difficulty with self-management activities, especially lifestyle modifications; (2) hesitancy intensifying medications by both clinicians and patients; (3) varying the timing and follow-up after changes in medication; (4) variation in blood pressure self-monitoring recommendations and uptake; and (5) limited specific functionality of current health information technology tools.

**Conclusions and Relevance:**

In this qualitative study of the views of PCPs and patients on hypertension control, the participants felt that more focus should be placed on lifestyle modifications than medications for hypertension, particularly for patients from racial and ethnic minority groups. Participants also expressed concerns about the existing functionality of health information technology tools to support increasingly asynchronous hypertension care. More intentional ways of supporting treatment intensification, self-care, and follow-up care are needed to improve hypertension management for racially and ethnically diverse populations in primary care.

## Key Points

**Question:**

What are patients’ and clinicians’ perspectives on hypertension control and management in racially and ethnically diverse populations in primary care?

**Findings:**

In this qualitative study of 15 patients and 15 clinicians, the participants felt that self-management (in particular, lifestyle modifications) should receive more attention; for example, they expressed difficulty with self-management activities, especially lifestyle modifications, and some hesitancy about and variation in intensifying medications and recommendations for follow-up care and patient self-monitoring. Current health information technology tools for hypertension were also thought to be limited.

**Meaning:**

The results of this study suggest that, as current care models for hypertension are thought to be insufficient, more attention may need to be paid to ways to support treatment intensification and self-management, particularly through asynchronous interventions.

## Teaser

This qualitative study of 15 patients and 15 clinicians examinged the barriers and facilitators to hypertension control within a racially and ethnically diverse health care system.

## Introduction

More than 100 million individuals in the US are estimated to have hypertension, and the rates of control remain suboptimal.^1^ Control of hypertension among Black and Hispanic or Latino patients lags even further behind.^2^ These disparities are problematic because of differences in consequences^3^; an increase of 10 mm Hg in systolic blood pressure is associated with an 8% increase in stroke risk among White patients but a 24% increase among Black patients.^4^

Of the many factors associated with hypertension, suboptimal adherence to antihypertensive medications and lifestyle modifications, such as diet and exercise, are thought to be central,^5^ and clinical inertia, where clinicians do not intensify treatments when indicated, occurs in up to half of treatment episodes.^6,7^ Suboptimal adherence and clinical inertia are more common among patients from racial and ethnic minority groups.^5,6,8^ Furthermore, effective blood pressure management relies on monitoring response to therapy, but gaining access to accurate blood pressure values is challenging; Black and Hispanic or Latino patients are less likely than White patients to receive follow-up care or use home self-monitoring devices.^2,9^

Current care models for managing hypertension appear to be insufficient.^10^ Given the increasing prevalence of hypertension,^11^ the shortage of primary care providers (PCPs),^12^ and the increasing interest in asynchronous care or care outside of offices (associated in part with the COVID-19 pandemic),^10^ there is a need to design effective strategies to efficiently improve the quality of hypertension care. To our knowledge, few studies have explored perspectives on what would be specifically helpful to improve hypertension care, especially among racially and ethnically diverse populations in the US.

We sought to examine the barriers and facilitators to efficient and effective hypertension care using in-depth qualitative interviews with patients and PCPs. We also sought to explore specific perspectives on current hypertension health information technology tools to recommend interventions that may overcome barriers.

## Methods

This study, conducted between October 1, 2020, and March 31, 2021, followed the Consolidated Criteria for Reporting Qualitative Research (COREQ) guidelines.^13^ Patient and clinician participants provided verbal consent to participate and for use and recording of their interviews for research purposes, including publication. This study was approved by the Mass General Brigham institutional review board.

Participants were recruited from Advocate Aurora Health, a large integrated health care delivery network in Illinois and Wisconsin serving a racially and ethnically diverse population. We used electronic health records (EHRs) to identify patients meeting the following eligibility criteria: (1) aged 18 years or older, (2) hypertension diagnosis, and (3) PCP visit at a study clinic in the prior 2 years. We focused on 2 Chicago clinics that serve a racially and ethnically diverse patient population to avoid contamination with a subsequent trial.^14^ Clinicians excluded patients if they thought they could not participate for cognitive reasons. Eligible patients were invited by telephone and an EHR-linked patient portal to participate. Potentially eligible clinicians included family practice and internal medicine primary care physicians, PCP-designated nurse practitioners, and medical assistants, who were contacted by email.

### Interviews

To elicit personal accounts about hypertension management, we used individual, semistructured qualitative interviews. The lead author (J.C.L.), who is experienced in qualitative methods, drafted comprehensive guides that were designed to build empathy and that were iteratively refined by other coinvestigators (R.A.B., R.K., N.G., A.S., M.A.A., P.A.K., L.S., K.H., N.H., and N.K.C.) with expertise in primary care, behavioral science, disparities, and cardiology. Constructs from the Consolidated Framework for Implementation Research informed the development of the interview guides. We pilot tested and finalized guides with nonparticipant volunteers. The patient guide focused on coping with (ie, managing) high blood pressure, including adherence to medication and lifestyle modification, perspectives of monitoring tools, experiences accessing resources and tools, and interactions with clinicians about hypertension (eTable 1 in Supplement 1). The clinician guide focused on current practice and the challenges of managing patients with hypertension, considerations for initiating and intensifying medications, experiences using the EHR and other tools for hypertension, and suggestions for improvement (eTable 2 in Supplement 1).

Due to the COVID-19 pandemic, interviews were conducted virtually using the Zoom video and audio platform and audio recorded. Interviews were conducted by a trained moderator and pharmacist (J.C.L.) in English. Several strategies were used to minimize misperceptions, including emphasizing that they were not part of the medical team. Before the interview, verbal consent was attained from clinicians and patients for permission for audio recordings. Clinicians were asked brief questions about race and ethnicity, sex, and training; information about race and ethnicity, age, sex, and blood pressure medications and values was obtained from the EHR for patients. The interviewer followed the guide but modified questions and asked follow-up questions depending on the participant’s responses.

Sequential interviews were conducted until reaching thematic saturation.^15^ Each interview lasted 20 to 60 minutes (mean [SD] duration, 38 [14.1] minutes). Patients were offered $50 as compensation; clinicians’ clinics received $150.

### Analysis

Analysis was conducted between June 2021 and February 2022. Interviews were transcribed verbatim and checked for accuracy. Two investigators (J.C.L. and R.A.B.) annotated a transcript selection independently and devised preliminary codes; after discussion, these codes were revised and agreed on. Then, each transcript was analyzed by the same 2 investigators using immersion and crystallization methods.^16,17^ We continued to review cycles until all data were examined and meaningful patterns emerged. Preliminary coding revealed themes around (1) access to ambulatory self-monitoring, (2) individual ways of intensifying treatments and monitoring, (3) difficulty with diet and exercise, and (4) the limited functionality of EHR hypertension tools. Dedoose, version 8.3.47b (SocioCultural Research Consultants) was used for storage and handling and analysis.

## Results

We conducted 30 interviews with 15 patients (mean [SD] age, 58.6 [16.2] years; 10 women [67%] and 9 Black patients [60%]) and 15 clinicians (14 PCPs, and 1 medical assistant; 8 women [53%]) (Table 1). Fourteen patients (93%) were prescribed an antihypertensive medication; 11 (73%) had suboptimally controlled blood pressure (ie, latest EHR-recorded blood pressure ≥140/90).

**Table 1.** Characteristics of Participants

Characteristic	No. (%)
**Clinicians**	
No.	15
Clinical role	
Physician	13 (87)
Medical assistant	1 (7)
Nurse practitioner	1 (7)
Gender	
Female	8 (53)
Male	7 (47)
Race and ethnicity^a^	
Asian	2 (13)
Black	2 (13)
Hispanic or Latino	2 (13)
White	7 (47)
**Patients**	
No.	15
Age, y	
30-49	4 (27)
50-64	5 (33)
≥65	6 (40)
Gender	
Female	10 (67)
Male	5 (33)
Race and ethnicity	
Asian	1 (7)
Black	9 (60)
Hispanic or Latino	1 (7)
White	5 (33)
Latest blood pressure, mm Hg	
≥140/90	11 (73)

^a^ Two clinicians chose not to provide this information.

Patients reflected on how they contextualize their diagnosis and integrate hypertension care into their routines and their relationship with PCPs. Clinicians described how they manage patients’ hypertension, communicate with patients, and use the EHR. We identified 5 themes (Table 2). Each theme is presented with representative quotations; other quotations are in eTable 3 in Supplement 1.

**Table 2.** Summary of Key Themes Relevant for Primary Care

Theme	Key takeaways
Difficulty with self-management activities, especially lifestyle modifications	Patients report more difficulty with diet and physical activity, exacerbated by COVID-19 pandemic Clinicians also believe that adherence to medication is less of a challenge in their population
Hesitancy intensifying medications by both clinicians and patients	Thought to be more difficult to intensify medications than to initiate treatment Tend to individualize treatment choices Management typically done by primary care provider
Varying timing and follow-up after changes in blood pressure medication	Patterns of follow-up range reasonably widely even by clinicians within the same clinic Patient portal sometimes used to support follow-up
Variation in blood pressure self-monitoring recommendations and uptake	No consistent ways of recording or sharing of out-of-office blood pressure values Insurance key barrier to access Size of blood pressure cuff represents an important physical barrier
Limited specific functionality of current health information technology tools	Interest in better way to observe longitudinal trends in hypertension care (eg, values and medications) Largely use self-created patient education materials because of lack of preferred electronic health record–embedded ones

### Difficulty With Self-management Activities, Especially Lifestyle Modifications

Patients acknowledged that maintaining a healthy lifestyle is very difficult; even among those who used strategies to adhere to a healthy lifestyle, adherence to those strategies was imperfect. Clinicians noted specific challenges that some patients face when making lifestyle decisions, including easy access to healthy food and exercise locations; structural barriers were exacerbated by the COVID-19 pandemic:

Patient: “Food and diet is a hard thing to do, especially with my kids—’cause if you change one person, you have to change everybody.”

Clinician: “I see people sometimes working 2 jobs trying to make ends meet, and so they find themselves sleeping maybe 4 [or] 5 hours. Then they have no time, which would help them with their blood pressure.”

Clinicians and patients alike generally believed that medication adherence was less difficult than lifestyle change. This belief stemmed from the fact that most antihypertensive medications are inexpensive and thus access would be easy. Clinicians described patient confusion about which lifestyle choices are truly healthy, recognizing that there may also be ranges of recommendations for patients, while medication-taking was deemed more straightforward. Some patients also reported clear medication-taking routines:

Patient: “I just sit the pill bottle on the table, so as I’m getting ready for work, I make sure I take my medicine.”

Clinician: “We’re an inner-city practice, and so cost is always important, but honestly, all those meds are generic. Really, the biggest barriers are, for my patients, lifestyle, and they don’t feel sick. In the south side of Chicago, I feel like that’s the biggest barrier because there’s pharmacies all over the place.”

Regardless, for some patients, taking medications was their least favorite part of their hypertension care, even though it may not be as difficult for them:

Patient: “I’m one of those people, don’t just give me pills. Give me some things to do. I don’t want to take all those pills if I don’t have to.”

Patient: “I hate taking medicine. I travel a lot with friends, and it’s so funny because we go to breakfast, and we’re a bunch of old ladies pulling out these little pill bottles—I hate it.”

### Hesitancy Intensifying Medications by Both Clinicians and Patients

Another common topic was intensifying antihypertensive medications. Patients often preferred sticking with lifestyle modifications and/or 1 medication, in the hopes of achieving blood pressure control. Clinicians felt that it was frequently more difficult to have conversations about additional medication than starting medication initially, in part because they feared pushback or losing engagement altogether:

Patient: “He’s [doctor] been tryin’ to get me to change that medication for some time. I just was really tryin’ to get off of it before it’s time to go to another one.”

Clinician: “It depends on age. If they’re young or elderly, it’s, ‘I don’t want be on a medicine.’ If in-between, sometimes there’s more toleration, but we’ll try something, they’ll get scared, won’t like a side effect, and then they’ll give up and I’ll lose them to follow-up.”

When the choice to intensify treatment is made, clinicians provided several rationales when choosing treatments, although they relied largely on national guidelines, with some variations based on the perceived strength of evidence. Clinicians also described how they personalize their prescribing, focusing mainly on comorbidities or adverse effects when prescribing rather than factors such as sex or race and ethnicity:

Clinician: “I don’t go with that whole Black man, White woman thing. For elderly, I’m going to use the same medication but lower doses. I try to avoid diuretics in older women, so they don’t go leaking urine all over.”

Clinician: “You want to make sure that you’re optimizing someone’s therapy for that person. What medication you choose definitely matters depending on race, ethnicity, comorbidities. It’s never the same for everyone.”

### Varying Timing and Follow-up After Changes in Blood Pressure Medication

Many patients expressed a preference for clinic-based interactions for hypertension, worrying that their connection with clinicians is otherwise insufficient. Some clinicians described specific approaches for follow-up, while others’ decisions were based on their perception of patients’ likelihood of following that plan. Follow-up patterns varied even by clinicians within the same clinic:

Patient: “I had one virtual meeting, and after that, I said, ‘Hey, doc. I don’t like this. Next visit, I want to see you in person.’ Because he’s sitting right there. I can look him in the eyes. He can check my heartbeat and all that.”

Clinician: “Some of it depends if it is a patient that is going to monitor their blood pressure at home. I’m usually aiming for like, 10 to 20 different readings, however they want to do that. If they don’t use the portal, I often have my nurse or medical assistant follow up in 2 to 3 weeks to review their blood pressures.”

Clinicians had a modestly favorable view of the patient portal (ie, EHR-linked communication platform) to support follow-up but recognized that patient perceptions about and willingness to use the portal varied widely. Patients generally had strong reactions to the portal; some loved it, while others thought it was too complicated:

Patient: “The portal is really cool. You can schedule virtual visits from there too. If it’s an emergency, like with my blood pressure, I call that number and she calls right back.”

Patient: “I don’t like it. I’m not into all that computer, electronic stuff. I’m an old-school guy, and I’m not up-to-date on all this computer, electronic, media stuff now.”

Clinician: “Usually, I’d have them come back in their usual 3-month visit. If they have their own cuff and since it’s COVID, I’ll have them check their blood pressures over the next month and send me stuff over the portal.”

### Variation in Blood Pressure Self-monitoring Recommendations and Uptake

Patients and clinicians alike described variation in how often they used or recommended, respectively, home self-monitoring. Clinicians described varying success in obtaining values, including requesting patients to send them via the EHR portal, email, or telephone or requesting in-person blood pressure checks. Several clinicians recognized increased reliance on patient-reported data during the COVID-19 pandemic but found it difficult to direct care due to ranges in how data are collected. A common barrier was ensuring consistent or easy access to blood pressure monitors, particularly because clinicians had little knowledge about whether insurance would cover them. Clinicians expressed great interest in better guidance:

Patient: “I would if I had extra money. I know I need to do it. It’s just, when can I spare that extra money?”

Clinician: “So often, we ask patients to monitor at home, and you get a small chunk who will get a cuff and report data back. There is some confusion about the appropriate cuff. As a physician, I will, in our EHR, order a cuff, and I think, done. Right? Cuff’s been ordered. Well, was there coverage? There’s a lot of challenges I don’t even recognize about how one secures a cuff and if they secure an appropriate one. When patients come back and actually did A, B, and C, I think ‘Wow, you did?’”

Physical barriers, such as blood pressure cuff size, were also repeatedly mentioned by patients and clinicians as something confusing. Standard cuffs were often too small for overweight patients. Patients also reported often preferring wrist cuffs, but clinicians vastly preferred arm cuffs, citing increased accuracy and data quality:

Patient: “I don’t really check it at home because it’s really hard to get a cuff. They don’t fit when I order a small cuff, and I need a bigger cuff. I do take my blood pressure, but I don’t think it’s always accurate.”

Clinician: “There’s such variability in the quality of cuffs available to the public, and some people buy a cuff that goes around your wrist, and I don’t trust those.”

### Limited Specific Functionality of Current Health Information Technology Tools

Most clinicians did not describe any specific health information technology tools for hypertension that they routinely used or liked, such as those available in the EHR system. Of the range of suggestions, the most common were an interest in better tracking and observing longitudinal trends in care (eg, systolic blood pressure values and medications) embedded in the HER:

Clinician: “I still have everyone giving me little pieces of paper I try to copy and get scanned. Then it goes into the media file, but I have to search. So, a clearer way to trend stuff, patients being able to input info easily that show me in a graph.”

Regarding mobile health (mHealth) apps or other patient-directed education, clinicians expressed concerns about the quality of information and rarely recommended patient-facing apps. Instead, they preferred to provide written educational material, such as background information about hypertension to add to EHR-based after-visit summaries, but they expressed uncertainty about whether patients used the material because it was either too voluminous or untailored. Consequently, PCPs often described using self-created materials. Patients wanted better information and reported minimal mHealth use:

Patient: “I wonder if information can be given to us as patients to help us to control our blood pressure and maybe eventually wean ourselves off medication. Something more proactive than writing a prescription.”

Clinician: “I would love a little more streamlined, ‘This is the highest level of evidence’ summary sheet for sharing patient information.”

## Discussion

In this qualitative study of patients and clinicians in a health system with a racially and ethnically diverse patient population, participants described a range of perspectives and experiences with hypertension care in primary care. Supporting patients with lifestyle modifications, blood pressure self-monitoring, and treatment intensification appeared to be key areas for potential improvement. Participants also expressed concerns about the functionality of health information technology to support asynchronous hypertension care, suggesting the need to design more effective strategies to efficiently improve quality within current constraints.

In the US, most studies have focused on hypertension beliefs among racially and ethnically diverse patient populations rather than specific management practices, but they have elicited similar themes about wishing to avoid medication and preferring PCPs who focus on lifestyle modifications.^18,19^ Prior research also suggests that many patients, particularly in Black communities, expect hypertension to be cured.^20^ Other non-US studies have identified similar themes. Interviews in the Netherlands observed that patients did not prioritize hypertension and were interested in deprescribing antihypertensives.^21^ Clinicians in Australia indicated hesitancy about intensifying medications, especially for older adults.^22^ Patients with hypertension in Canada expressed similar interest in lifestyle options rather than medications.^23^ To our knowledge, few studies have focused on clinician and patient barriers to efficient care.

These findings hold important implications for the design of interventions to improve blood pressure management. First, patients and clinicians believed that they are more challenged by lifestyle modifications compared with medication taking, although evidence suggests that adherence to antihypertensive medications is still suboptimal.^24,25^ Many noted that some lifestyle challenges were more prominent in Black and Hispanic or Latino communities. Thus, to be most acceptable to patients, adherence interventions may need to incorporate lifestyle recommendations to improve management.^26,27^

Second, blood pressure self-monitoring remains a source of confusion. Increasing interest in home-based hypertension strategies is underscored by inherent challenges in office-based hypertension management.^10^ Some effective options include interventions combining home blood pressure monitoring with medication titration protocols, although these interventions have not been extensively studied in racially and ethnically diverse communities.^28^ To enact these strategies, our study highlights some additional logistical challenges, including streamlining access to blood pressure cuffs, ensuring that PCPs and patients can obtain the most appropriate cuff for arm size, and value integration. Care should also be taken to ensure that devices are affordable, even though costs have been decreasing in recent years. Rates of blood pressure self-monitoring may also be lower in Black and Hispanic or Latino populations, due to differences in both how often self-monitoring is recommended by clinicians and how often it is done by patients.^29^

Third, gaps in treatment intensification were repeatedly described. Systematic medication titration protocols may help address this issue, but extensive study in multiethnic populations remains limited.^30^ Thus, interventions addressing concerns around treatment intensification, especially when increasing doses or adding new medications, seem necessary to address hesitancy. One avenue could be improving how EHR systems present longitudinal trends to illustrate need.^31^

Finally, gaps may also exist between the availability of patient-facing health information technology and uptake. Emerging literature remains mixed about mHealth apps and text messaging and lowering blood pressure^32^; these interventions appear most effective with 2-way communication with health care professionals.^33,34^ They have also been minimally studied in diverse populations.^35^ Our study suggests an implementation gap between their availability and their uptake, warranting further study.

### Limitations

This study has several limitations. First, it was conducted in 2 clinics and with only 30 participants (although saturation was reached), which may limit generalizability, and further evaluation may be necessary. Second, while interviews were conducted by an external interviewer, some response bias may still be possible in response to the questions, especially because the interviewer was a pharmacist. Third, clinicians could exclude patients from interviews but focused on excluding those with cognitive difficulty. Fourth, interviews were conducted during the COVID-19 pandemic but should be generalizable to ongoing combinations of in-person and asynchronous care.

## Conclusions

Participants in this qualitative study had heterogeneous experiences with hypertension care, even within the same clinic, and felt that more focus should be on lifestyle modifications. More intentional and tailored ways of supporting treatment intensification, self-care, and follow-up care may be needed to improve hypertension management in primary care practices serving diverse populations.

## Notes

Supplement 1. **eTable 1.** Overview of Semi-Structured Guide for Patients **eTable 2.** Overview of Semi-Structured Guide for Providers **eTable 3.** Additional Supporting Quotations Relevant for Each Theme

Supplement 2.
Data Sharing Statement
