# Supplementary material for: Clinicians’ and Patients’ Perspectives on Hypertension Care in a Racially and Ethnically Diverse Population in Primary Care
Source: JAMA Netw Open. 2023 Feb 28;6(2):e230977. doi: 10.1001/jamanetworkopen.2023.0977 (PMC9975920; doi:10.1001/jamanetworkopen.2023.0977)
Supplement: Supplement 1. — eTable 1. Overview of Semi-Structured Guide for Patients eTable 2. Overview of Semi-Structured Guide for Providers eTable 3. Additional Supporting Quotations Relevant for Each Theme [file jamanetwopen-e230977-s001.pdf]

Supplementary Online Content

Lauffenburger JC, Barlev RA, Khatib R, et al. Clinicians' and patients' perspectives on hypertension care in a racially and ethnically diverse population in primary care. *JAMA Netw Open.* 2023;6(2):e230977. doi:10.1001/jamanetworkopen.2023.0977

eTable 1. Overview of Semi-Structured Guide for Patients
eTable 2. Overview of Semi-Structured Guide for Clinicians
eTable 3. Additional Supporting Quotations Relevant for Each Theme

This supplementary material has been provided by the authors to give readers additional information about their work.

© 2023 Lauffenburger JC et al. *JAMA Network Open.*

eTable 1. Overview of Semi-Structured Guide for Patients

Key Areas	Semi-structured interview guide
Introduction	• Approximately how long ago did you find out that you had high blood pressure? Were you surprised? (Why or why not?)
Coping with blood pressure	• What do you do to cope with your high blood pressure? Does your blood pressure stay the same or does it change?<br>• We know that you know that eating healthy is important, but not everyone has the ability to buy healthy food or cook healthy foods. What works for you? What doesn't work for you?<br>• Just like eating healthy, being physically active is important to your health. When are times that are easier for you to be physically active and why? When are times that are harder for you?<br>• How many medications do you take for your blood pressure? Do you know what they are? How regularly are you able to take them?<br>• How does your blood pressure medication make you feel?
Perspectives and barriers to monitoring tools	• People sometimes have difficulty figuring out what their blood pressure is going to be. What blood pressure monitoring tools do you have access to, either at home or in your community?<br>• One reason people have not been monitoring their blood pressure is accessing this. What barriers come to mind when you consider accessing or being able to afford blood pressure monitoring?<br>• What issues have you found when monitoring your blood pressure?
Resources and other tools	• Do you feel like you are able to get access to the resources you need to monitor your blood pressure at home (or not in the office)?<br>• COVID-19 has changed a lot of things for some people; for others it has not. How has COVID-19 changed how you cope with your blood pressure?<br>• How often do you use the Patient Portal? What do you like and dislike about it? If you haven't used it, why?<br>• What other tools do you use to manage your blood pressure?
Provider and healthcare system interactions	• How was your last visit with your doctor? How much did you discuss your blood pressure?<br>• Do you remember talking to your doctor about blood pressure or medications? How about food or exercise? Do you remember how it came up? Were there any discussions about other aspects of blood pressure treatment other than medication?<br>• Have you ever been referred to or worked with a social worker/patient navigator for your blood pressure? What about for anything else?<br>• If you need to contact your doctor outside of an office visit, how do you reach them? How do they respond when you reach out?

© 2023 Lauffenburger JC et al. *JAMA Network Open.*

eTable 2. Overview of Semi-Structured Guide for Providers

Key Areas	Semi-structured interview guide
Introduction	• How many years have you been practicing?<br>• How long have you been working at Advocate?
Current practice and challenges	• How do you manage typical patients with hypertension? Which guidelines do you typically use?<br>• What challenges do you most experience when managing patients with hypertension (assuming established diagnosis)?<br>• How do you manage patients with persistently poorly controlled hypertension? Can you give us an example of a recent patient you worked with?<br>• What challenges are often reported by patients about how they manage their own blood pressure? How do you discuss challenges regarding access or affordability of management tools with your patients?
Initiation and intensification of medications	• Tell me about the last time you wrote a new blood pressure prescriPatiention or changed a patients' dose. What do you typically recommend now for managing patients initiating or titrating their therapies?<br>• How do you figure out whether the medication worked? How do you determine whether patients are adherent to their medications? What workarounds do you use to monitor your patients after your visit?
Clinic practice/use of EHR tools	• Walk me through a *typical day* in how you use the electronic health record system. How often do you check the in-basket? When do you open an encounter or the chart relative to a patient coming in for an office visit? Do you open the chart while the patient is there? Say you want to intensify medication, when do you write the order?<br>• What challenges do you experience when using the EHR?<br>• What does communication with patients look like outside of scheduled office visits?<br>• How has COVID-19 changed the way you manage patients with hypertension?
Solutions to barriers	• We have discussed several barriers to caring for patients with hypertension. What EHR tools do you think could be improved upon in order to address some of these barriers?<br>• What could be done to improve communication with patients and staff?<br>• What information or tools would be helpful for you as a provider to ensure proper care for patients with hypertension?<br>• Do you encourage the use of the portal? How could the patient portal be improved?<br>• Consider after-visit summaries for patients. What information would best serve your patients with hypertension on these summaries?

Abbreviations: EHR, electronic health record

© 2023 Lauffenburger JC et al. *JAMA Network Open.*

eTable 3. Additional Supporting Quotations Relevant for Each Theme

Theme	Additional supporting quotations
Difficulty with self-management activities, especially lifestyle modifications	• Patient: “I’m good at getting started with exercising, but staying consistent and doing the frequency of it, that’s where I fall short.”<br>• Patient: “It’s really hard to make different meals. I seem to be eating basically the same things all the time, which gets boring.“<br>• Clinician: “Many patients in these populations have extra problems because of also having a lot of stresses and anxieties and depression, all the family issues they are going through and how it impacts them.”<br>• Clinician: “Taking pills is relatively easy compared to doing lifestyle interventions, right? How do you get people to change how they're living their lives to maybe improve the blood pressure? There's so much focus on the pills.”<br>• Clinician: “Our patients are not taking time to take care of themselves. Get outside, exercise. Focus on how they eat. The obesity pandemic is as severe as the coronavirus in some ways.”<br>• Clinician: “Telling people how to eat healthy is a whole other animal. If all my hypertensive patients lost weight or exercised more often, I’d have fewer patients.”
Hesitancy intensifying medications by both providers and patients	• Patient: “A first, I didn’t wanna be on the pills. I told my doctor, ‘I will start taking them, but I’ll find a way to get rid of them. Now I have to take two pills and really don’t like it.’”<br>• Clinician: “I double the dose first; refill with 25 milligrams and come back in four weeks.”<br>• Clinician: “You say, ‘We’ll bump you up from 10 to 20mg and, if that doesn’t work, we’ll go to 40,’ but you won’t see them again until next year, or maybe you’ll see them for some other reason but, when they come in because they sprained their ankle, you aren’t likely to actually make the change, even though you’re aware we’re not at goal.”<br>• Clinician: “For a lot of my patients, adding on more medicines is a challenge, which I’ve never really understood. Even a combo pill; it’s that they are resistant to adding more medicine ‘cause they want to be as ‘natural as possible’.”<br>• Clinician: “I feel like we’re potentially limiting ourselves and narrowing oPatientions. If all Black patients are supposed to be on diuretics, and they hate them, they’ll eventually not take them.”
Varying timing and follow-up after changes in blood pressure medication	• Patient: “I rely on the doctors to give me the blood pressure pill. I take it religiously, so I haven’t been monitoring at home. I used to monitor but now I don’t; my vision is also so low that I can’t read printouts well.”<br>• Clinician: “I've gotten more aggressive with prescribing once I shifted into attending-hood and pandemic. Say like, ‘I'm gonna send you a cuff.’ I haven't had a huge amount of success with that either because of insurance.”

© 2023 Lauffenburger JC et al. *JAMA Network Open.*

**Theme**	**Additional supporting quotations**
Variation in blood pressure self-monitoring recommendations and uPatientake	• Clinician: "It doesn't do as much good if patients are checking at home and it's consistently in the 160s and nothing gets done."<br>• Patient: "Me not having a larger cuff is a really big issue 'cause I can't take my blood pressure at home. If they could help me better navigate one either through insurance or fairly cheaply, that would be awesome."<br>• Clinician: "A barrier I've noticed for my patients who are morbidly obese, they go and try and get an arm cuff, and they're like, "It won't even fit." I don't know brands that fit a larger arm, and to be honest, I don't even know what the max circumference is for most monitors."<br>• Clinician: "Even in the clinic, we used to have just the large-regular, but now with the large-long, it makes a huge difference."
Limited specific functionality of current health information technology tools	• Patient: "Some doctors give you stuff and don't even explain it."<br>• Clinician: "There ends up being a lot of info in the after-visit summary. It's like when a patient leaves the hospital and have this huge packet they never look at."<br>• Clinician: "People don't want to read. If you get a snapshot or do a little video, people would pay attention to that rather than paper."<br>• Clinician: "A cool hypertension dashboard where I would see all of the trends because, at least with our EHR, once I'm in an active encounter, I can see maybe the last three readings."<br>• Clinician: "Especially in COVID, it might be good to have something where you can say, 'in the past three weeks', like a timeline with values."

© 2023 Lauffenburger JC et al. *JAMA Network Open*.
