# Supplementary material for: Clinicians’ and Patients’ Perspectives on Hypertension Care in a Racially and Ethnically Diverse Population in Primary Care
Source: JAMA Netw Open. 2023 Feb 28;6(2):e230977. doi: 10.1001/jamanetworkopen.2023.0977 (PMC9975920; doi:10.1001/jamanetworkopen.2023.0977)
Supplement: Supplement 2. — Data Sharing Statement [file jamanetwopen-e230977-s002.pdf]

Data Sharing Statement

Lauffenburger. Clinicians' and Patients' Perspectives on Hypertension Care in a Racially and Ethnically Diverse Population in Primary Care. *JAMA Netw Open*. Published February 28, 2023. doi:10.1001/jamanetworkopen.2023.0977

Data
Data available: No

Additional Information
Explanation for why data not available: Data will be available upon reasonable request and through execution of any appropriate data use agreements, or agreements deemed necessary by Mass General Brigham and Advocate Aurora Health.
